# The Binary-Based Model (BBM) for Improved Human Factors Method Selection

**DOI:** 10.1177/0018720820926875

**Published:** 2020-06-18

**Authors:** Matt Holman, Guy Walker, Terry Lansdown, Paul Salmon, Gemma Read, Neville Stanton

**Affiliations:** 1 3120 Heriot-Watt University, Edinburgh, UK; 2 5333 University of the Sunshine Coast, Queensland, Australia; 3 7423 University of Southampton, UK

**Keywords:** HF methods, method selection, fuzzy logic, complexity

## Abstract

**Objective:**

This paper presents the Binary-Based Model (BBM), a new approach to Human Factors (HF) method selection. The BBM helps practitioners select the most appropriate HF methodology in relation to the complexity within the target system.

**Background:**

There are over 200 HF methods available to the practitioner and little guidance to help choose between them.

**Method:**

The BBM defines a HF “problem space” comprising three complexity attributes. HF problems can be rated against these attributes and located in the “problem space.” In addition, a similar HF “approach space” in which 66 predictive methods are rated according to their ability to confront those attributes is defined. These spaces are combined into a “utility space” in which problems and methods coexist. In the utility space, the match between HF problems and methods can be formally assessed.

**Results:**

The method space is split into octants to establish broad groupings of methods distributed throughout the space. About 77% of the methods reside in Octant 1 which corresponds to problems with low levels of complexity. This demonstrates that most HF methods are suited to problems in low-complexity systems.

**Conclusion:**

The location of 77% of the rated methods in Octant 1 indicates that HF practitioners are underserved with methods for analysis of HF problems exhibiting high complexity.

**Application:**

The BBM can be used by multidisciplinary teams to select the most appropriate HF methodology for the problem under analysis. All the materials and analysis are placed in the public domain for modification and consensus building by the wider HF community.

## Introduction

### An Inconvenient Truth

Recent Human Factors (HF) literature converges on a core idea. The rate at which the HF discipline is moving beyond analyses of individual people interacting with individual artifacts to increasingly focus on complex systems problems, involving many people and many artifacts, is outpacing the uptake of existing systemic methods and the development of new ones (Salmon, Walker et al., 2017; Walker et al., 2017; Walker, Stanton, Salmon et al., 2010; Woods & Dekker, 2000). As a result of this, the limitations of reductionist methods are becoming exposed (Stanton et al., 2014; Stanton & Harvey, 2017; Trapsilawati et al., 2015; Walker, Stanton, Salmon et al., 2010; Young et al., 2015). Reductionist methods, which currently dominate the HF field (e.g., Walker, Stanton, Salmon et al., 2010), focus on reducing systems down to their component parts. They operate under an assumption that if the individual components can be understood, then so can the overall system which they comprise (Walker et al., 2016). This is a rather simplistic characterization and, to the extent it holds, an entirely appropriate one for many classes of HF problem. Indeed, it has driven the discipline forward for 50 or more years. While appropriate for some classes of HF problem it is not suited to others (e.g., Lee, 2001; Sharma, 2006), particularly the “high-quality” strategic problems the discipline needs to tackle in order to remain relevant (Dul et al., 2012): problems such as profound changes in global work systems, new developments in information and communications technology, the fourth industrial revolution, sustainability, and many more. These strategic problems are characterized as “systems problems” (Dul et al., 2012; Salmon, Walker et al., 2017; Wilson, 2014) and a growing body of recent research highlights the weakness of reductionist methods for tackling them (Salmon, Walker et al., 2017; Walker, Salmon, Bedinger et al., 2016, Walker, Stanton, Salmon et al., 2010; Woods & Dekker, 2000). This presents the discipline with an uncomfortable and disconcerting truth. The continued use of reductionist methods could potentially undermine HF practitioners’ ability to contribute to complex sociotechnical systems—the very systems they are increasingly being asked to work within (Walker, 2016), and for which the discipline itself sets its strategic future direction (Dul et al., 2012).

### Complexity and a Contingent Approach to HF Method Selection

Earlier work by the authors (Walker, Stanton, Salmon et al., 2010) provides a telling indication of the discipline as a whole. “Complexity” is a term that first occurred in the title of a paper in this journal in 1958 (Dean Chiles, 1958). Since then over 80 papers have featured either the word “complex” or “complexity” in their titles. *“Out of those 80 papers, nearly 90% have been published since 1990”* (p. 1175). The key issue is not whether the traditional domains of HF have become more or less complex. It is that previous successful HF interventions have increasingly exposed the human performance variability residing within this complexity. For example, in many domains safety improvements have exposed new classes of problem featuring a prominent human dimension (e.g., Civil Aviation Authority [CAA], 2011; Coury et al., 2010; European Aviation Safety Authority [EASA], 2010; Rail Safety & Standards Board [RSSB], 2009) and safety performance overall is plateauing (Viano et al., 1991). In some domains it is actually worsening (Bureau of Infrastructure, Transport and Regional Economics (BITRE), 2018). The methods that have yielded the prior improvements in performance are less effective with those problems which remain: the complex ones. This, in turn, creates the corresponding pressure on the discipline to find these new margins and address them (Salmon, Walker et al., 2017; Stanton, Salmon & Walker, 2017).

The definition of complexity, as applied to HF, is that a particular HF problem would consist of “several parts” and be “involved, intricate or difficult” in some way (e.g., Allen, 1984, p. 145; Richardson et al., 2004). Formal definitions of complexity vary depending upon whether complexity attributes, theory, or adaptive systems viewpoints are being adopted. These are discussed in depth in Walker, Stanton, Salmon et al. (2010) and summarized in Table 1.

**Table 1:** Definitions of Complexity

Label	Short definition
Attribute view (AV)	Complex systems can be characterized by the extent to which they embody (qualitatively) a common set of features such as uncertainty, dynamism, and multiplicity.
Complexity theory (CT)	Complex systems can be characterized by a quantitative measure, a single number, or an algorithm, which specifies a system. This is typically based on Kolmogorov complexity (*K*) and the ability of an object (*w*) to be represented by a given number of data bits (*x*). If the object is random, and therefore has a high rate of entropy (*h*_µ_), then the shortest algorithm which would produce *w* would be equal to the length of *w* itself. The less entropy and randomness (*h*_µ_) there is in the object (*w*) being described, the shorter the algorithm describing *K*_*x*_ can be.
Complex adaptive systems (CAS)	Complex systems can be characterized as “emergent behavior exhibited by interacting systems operating at the threshold of stability and chaos” (Roetzheim, 2007, p. 4); “systems with a large number of interacting parts and a large throughput of energy, information, or material” (Hübler, 2005, 2006; 2007, p. 10); and systems that “don’t just passively respond to events [..] they actively try to turn whatever happens to their advantage,” which is to say they are “adaptive” (Waldrop, 1992, p. 11).

As has been argued previously in Walker, Stanton, Salmon et al. (2010), Walker, Salmon, Bedinger et al. (2016), and Walker (2016), complexity is a powerful contingency factor when selecting appropriate HF methods to apply in practice. In other words, HF methods need to be matched to the levels of complexity inherent in the problem space in order to ensure high levels of predictive efficiency (PE; Crutchfield, 1994). PE is given by

$$PE=\frac{h_{\mu }}{K_{x}}$$

where *h*_µ_ is a measure of “entropy” or the amount of randomness contained in a system. Entropy can be measured in a variety of ways. A very simple example is to base it on a comparison between the system behaviors predicted by an HF method compared to those behaviors actually observed (e.g., Stanton & Young, 1999a). Any disparity between “expected” and “observed,” and in what quantity, represents an approximation to randomness or *h*_µ_.

*K*_*x*_, on the other hand, is the Kolmogorov complexity of the system’s model, in this case the HF method output, at any given scale of observation. This can also be measured in a variety of ways. One example is to base it on the number of “build symbols” in the system model (e.g., the number of functions contained in a Work Domain Analysis; Vicente, 1999), the sophistication of the model (i.e., the number of logical operators used in Hierarchical Task Analysis [HTA] plans; Stanton, 2006), or the model’s connectivity (the maximum number of links present in both), and so on.

These theoretical points connect to HF practice in a particularly vivid way. They encapsulate the familiar suspicion that one’s method outputs have become bigger and more complex than the system under analysis and that effort is being wasted compared to the level of insight being generated. What this actually describes is a situation of low PE where the cost, time, and resource implications of a “brute force” HF method are not repaid by value-adding insight. High PE is (or should be) the goal of all HF method applications, in theory and in practice. High PE is a case of the most parsimonious and efficient method being selected on the basis it provides the maximum of insight. Precisely this issue was explored in Walker, Salmon, Bedinger et al. (2016). In this study two teams were challenged to find weaknesses in a fictional complex sociotechnical system. Each team carried out a distinctive approach. One team used a reductionist approach, a Human Error–Hazard And Operability (HE-HAZOP) study (Kletz, 1974), and the other team used a systems approach, a WDA (Jenkins et al., 2009: Naikar, 2013; Rasmussen et al., 1994; Vicente, 1999). The team which employed the systems method which matched the systems problem at hand was able to find more weaknesses than the team which employed the reductionist approach. The PE of both approaches in finding system failures was modeled and the systems approach was found to be the most parsimonious for this particular problem. Although strictly illustrative, these findings highlighted two considerations central to the selection of HF methods more generally:

1. Selecting the method which can cope with the complexity inherent in the target problem under analysis
2. Selecting the method which maximizes PE

We argue that both of these issues are not given sufficient attention. Wickens et al. (2015) go so far as to judge that deciding appropriate methodology is perhaps the most problematic challenge encountered by HF practitioners today. The current paper aims to address this.

### The Evolution of HF Methods

**Figure 1:**
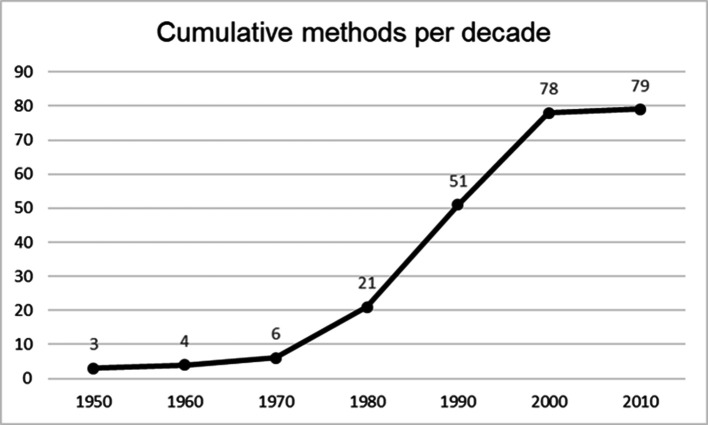
Cumulative development of methods per decade.

Whilst not being the principal aim of this paper, addressing the origins and evolution of HF methods over time is necessary to the present discussion. Exploration of the nascent forces, which both underlie the development of reductionist methods throughout the 20th century and perhaps explain their continued predominance, is long overdue. Holman et al. (2019) point out that 79 methods (Methods subject to this review were taken from *Human factors methods: a practical guide for engineering and design* by Stanton, Salmon, Walker et al., 2017. Only methods with discrete authors and dates of development were included in the review.) were developed between the 1950s—the dawning of the discipline proper—and 2010 (Holman et al., 2019; Figure 1). The relative stagnation of post-2000 method development is apparent. There may be several reasons for this. First, it is possible that these findings reflect a manifestation of quasi-Darwinist adaptation, whereby the conception of reductionist methods emanated from a sociotechnical (and perhaps intellectual) context which was largely described and understood in terms of cause and effect (Dekker, 2016). In this sense, their evolution throughout the latter half of the 20th century can be analogized to incremental adaptations to the prevailing contemporaneous sociotechnical paradigm. This argument can be extended to explain how it may be possible that the comparatively slower rate of technological advancement throughout the 20th century led to a pace of methodological advancement that was broadly commensurate with expectations; the relatively slow advancement of industrial technology simply afforded more time for methods to be iterated, modified, and adapted as needed (It should be noted that the term “advancement” refers to the sophistication, i.e., post-determinism, of methods and not the number of methods developed.).

A second possible explanation for methodological stagnation could be the prevailing engineering and safety risk management paradigm within which HF practice is often nested. This is particularly apparent in highly regulated high-hazard industries. In this context, a major advantage of reductionist methods (alluded to earlier) is their capacity for numerical output. Along with ease of communication and provision of “concrete” (and thus palatable) means of comparative analysis within the engineering context, numerical outputs allow practitioners to readily incorporate findings into wider probabilistic safety assessments. Whilst qualitative reductionist methods such as SHERPA and HE-HAZOP are broadly accepted within safety risk management (AS IEC 61882:2017; Health and Safety Executive, 2005), the practitioner still must engage in a potentially significant degree of inference in order to translate findings into concrete engineering outcomes.

Despite the recent stagnation in HF method development, some progress has been made toward integrating more sophisticated methods into formal HF, systems engineering and system safety practice. For instance, the inclusion of STAMP (Leveson, 2004) into recent systems engineering and system safety technical standards is particularly encouraging, as is the active and growing STAMP/STPA community. It is possible that these developments, among others, are an expression of the wider migration of the hitherto prevailing reductionist risk management and systems safety paradigm toward a more holistic systems approach (Altabbakh et al., 2014; Aven & Kristensen, 2005).

### Existing Approaches to HF Method Selection

There is an implicit guarantee in the use of HF methods that, provided they are used properly, they will produce certain types of useful products. Methods provide structure and the potential for repeatability. It has also been suggested that HF methods are a route to making the discipline more accessible to all (Corlett et al., 1995; Diaper, 1989; Stanton & Young, 2003). Given there are hundreds of HF methods to choose from (Stanton et al., 2013), and little explicit guidance on how to choose between alternatives, it is easy to understand why practitioners and organizations often develop their own or else find their favorites and continue to use them (Stanton & Young, 1998). The benefits of this pragmatism are clear. Familiarity with a particular methodology can cut costs, decrease training times within organizations, and enable smooth transfer of knowledge (Crawford et al., 2016). Over time, the continued application of particular methods creates a legacy. Within high-hazard industries, for example, regulations begin to specify them and further solidify their usage (e.g., BS EN 61882:2016). As a result, it is not uncommon for an organization to have just two or three commonly used methods often developed decades previously, despite the discipline having 200 or more, many of which are more up to date.

In account of the discussion above and the wider position put forward in the introduction, guidance on method selection would provide a concrete way for practitioners to help improve the HF “value proposition” (Dul et al., 2012) to external stakeholders and fulfill the strategic aims of the discipline going forward.

There are currently three broad ways in which HF methods are selected:

1. Legacy and preference;
2. Decision-support meta-methods;
3. Method integration/triangulation.

In the first case, developers of a particular method will establish its effectiveness (although there is often little hard evidence in the literature, see Stanton & Young, 1999a, 1999b; Stanton, 2016) and diffuse those benefits more widely, either through the academic literature and/or the provision of consultancy services. Many significant methods in the discipline follow this path, such as the Systematic Human Error Reduction and Prediction Approach (SHERPA; Embrey, 1983), the Cognitive Reliability and Error Analysis Method (CREAM; Hollnagel, 1998), the Functional Resonance Analysis Method (FRAM; Hollnagel, 2012), and the Situation Awareness Global Assessment Technique (SAGAT; Endsley & Kiris, 1995) to name just four. The advantage of this approach is that methods are made to leave the laboratory and be subject to the rigors of practical application. It is also common for methods to arise as a response to specific regulatory prerequisites (e.g., Directive 90/270/EEC). The disadvantage is that by inventing a particular approach, every problem begins to look like a suitable candidate. The further disadvantage is that some regulatory prerequisites may not be fit for purpose in a rapidly changing world.

**Figure 2:**
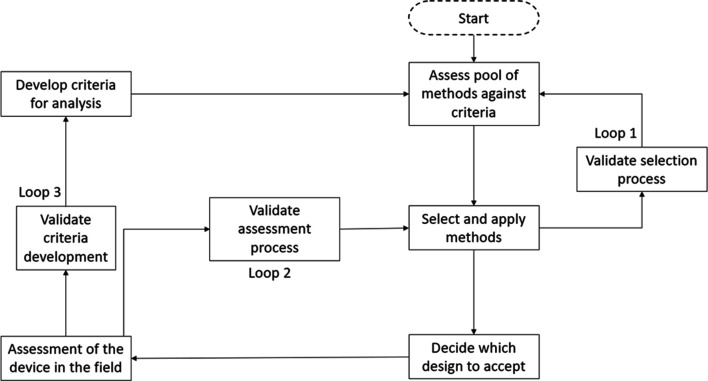
Adaptation of Stanton and Young’s HF method selection flow chart.

The second, and much less well-developed path, is the decision-support meta-method approach. This describes semiformal approaches used to match a wide(r) range of HF methods to a client or sponsor’s needs (e.g., Williams & Salmon, 2016; Wilson & Sharples, 2015). Stanton and Baber (1996) attempted to formalize this by putting forward a meta-method process based on establishing the reliability, validity, and utility of methods, which was latterly extended by Stanton and Young (1999a, 1999b) in the development of a decision-support flowchart shown in Figure 2. This presents method selection as a closed-loop process with three feedback loops. The first feedback loop validates the selection of the methods against the selection criteria. The second feedback loop validates the methods against the adequacy of the HF intervention. The third feedback loop validates the initial criteria against the adequacy of the intervention. Whilst this process appears to be logically sound, each step is open to interpretation and prone to misjudgment, especially when under time pressure. For example, there could be errors in the development of the initial criteria, the selection of the methods, and the appropriateness of the intervention.

The final approach is method triangulation and integration. Through triangulation, weaknesses in one method can be mitigated by the strengths of another (Corlett et al., 1995) and vice versa. Methods can be combined like this in any way which would suit the work system under analysis, so long as theoretical compatibility between the methods is ensured. Patriarca and Bergström (2017), for example, examined the utility of integrating the abstraction hierarchy from Cognitive Work Analysis (CWA; Rasmussen et al., 1994) with FRAM (Hollnagel, 2012) for analyzing risks associated with mooring a ship at a quay. The integration of these methods enabled additional analyses of inter-agent and intra-system level interactions. Houghton et al. (2015) triangulated the findings of social network analysis (SNA) and CWA in order to analyze military planning tasks. Triangulation enabled a more complete understanding of system structure, offering valuable insights. Similarly, Stanton (2016) combined CWA and SNA (see Scott, 1988) to compare dual, single, and distributed cockpit crewing for system safety on future flight decks. CWA was used to define the system constraints whereas the density statistic from SNA was presented as a measure of system resilience. Method triangulation reinforces the point that methods do not, nor do they have to, exist in isolation, a point developed by Salmon et al. (2005) in the form of an explicit methods matrix. The matrix enables methods to be clustered by domain of application, design stage at which they are appropriate, and whether one method is a necessary precursor for another. This high-level structure can be used to triangulate method outputs and strengthen the validity/reliability of subsequent findings. It can also be used to partner or integrate methods. An example of this is the Event Analysis for Systemic Teamwork (EAST) method which uses HTA, the Critical Decision Method (CDM), SNA, and information networks in an integrated manner (Stanton, 2014a; Stanton et al., 2019; Stewart et al., 2008; Walker, Stanton, Baber et al., 2010).

### Addressing the Method Selection Problem

The three approaches reflect the difficulties faced by practitioners in tackling the issue of appropriate method selection. We can attribute this to (or at least in part) there being no right or wrong way to approach method selection and application, particularly within the complex systems context (Salvendy, 2012). Even in the *Handbook of HF and Human Factors* (2012) Salvendy writes:

The most common answer to the appropriateness of a method for a given HF/E objective is: “*it depends*” (Salvendy, 2012, p. 299).

This paper, therefore, delves into the core of the clause “*it depends,*” and puts forward an approach to scrutinize and guide what method selection should, in fact, “*depend*” on. With this in mind, the paper introduces the Binary-Based Model (BBM) as a means of selecting HF methods in a structured and theoretically robust manner, but also in a way that is transparent and modifiable by the community at large. This is an attempt to continue a discussion on systems methods and the role of HF in addressing them.

## Binary-Based Model

The BBM provides a practical means for HF practitioners to select methods for maximum PE. The BBM is founded on the attribute theory of complexity (Walker, Stanton, Salmon et al., 2010) and a set of system thinking tenets (Grant et al., 2018), both described later. The guidance offered by the BBM, along with its capability to highlight where the discipline is lacking in methods, makes it a potentially powerful development. The underlying aims of the BBM are based on “product” and “process”:

- The product of the BBM—its output—is to encourage users to recognize the utility of systems-based methods in complex sociotechnical system analysis. This is concomitant with a broader aim of helping the community to investigate methods perhaps not previously considered and to broaden the methodological horizons of the discipline in general.
- The process of the BBM is equally important. It is a tool to help method users think more closely about issues of complexity and method matching, something that can help individuals and teams scrutinize their target HF problems in new ways.

The following sections describe the theoretical perspectives underlying the BBM, how they are used to drive the inputs to the BBM model, how the inputs are processed to produce measures of system complexity, and how these enable appropriate HF methods to be selected for a given problem.

## Theoretical Underpinnings

### The Attribute Theory of Complexity

The BBM is underpinned by the attribute theory of complexity. This theory posits that complex systems can be characterized by a common set of features. These features vary widely in their description. Woods (1988), for example, has proposed five key attributes: uncertainty, multiplicity, dynamism, importance, and difficulty. Cilliers and Spurrett (1999) suggest a bigger list, with complex systems having a large number of independent, dynamically interacting elements in an open system in which a constant flow of “energy” is required, alongside recurrent loops of interaction, path dependence, and nonlinearity. Whilst there is debate around the number of complexity criteria which are appropriate or useful, there are common themes. Walker, Stanton, Salmon et al. (2010) label these themes dynamism, uncertainty, and multiplicity.

Dynamism introduces the idea of changing system states and time spans as a feature of complexity. Broadly speaking, dynamism can be seen as the degree to which the nature of the system or problem can change over time, the degree to which the system can change states without intervention by an agent or user, and finally, the diversity of time spans for ongoing tasks within the system (Woods, 1988). Dynamism represents a challenge to methodological tradition wherein a static representation of human–system interaction is often assumed, as embodied by the ubiquitous psychology-inspired cross-sectional studies which analyze data from a population, or representative sample, at a specific point in time (Lee, 2001; Sharma, 2006). The dynamism attribute confronts the notion that not all HF problems possess the clinical levels of control found in the laboratory (e.g., Walker, Stanton, Salmon et al., 2010; Wilson, 2014) and there is often no “one right way” to perform a task (Howie & Vicente, 1998). Moreover, people will interpret their environment, “massage it and make such adjustments as they see fit and/or are able to undertake” (Clegg, 2000, p. 467). As Sharma (2006) states: “The human–machine system behaviour and performance are dynamic, nonlinear, and possibly chaotic” (p. 319). Furthermore, the tempo of this dynamism is, if anything, increasing (Woods & Dekker, 2000).

In complex sociotechnical systems, uncertainty manifests itself as limited knowledge of systemic components, their interconnections, and their potential to change over time (Cilliers & Spurrett, 1999; Walker, Salmon, Bedinger et al., 2017; Walker, Stanton, Salmon, Rafferty, et al., 2010; Walker, Stanton, Salmon et al., 2010; ). This makes it difficult to determine current system states and predict future states. HF research sometimes refers to this attribute (or elements of it) as “fuzziness” (e.g., Bell & Crumpton, 1997; Karwowski & Ayoub, 1984; Karwowski, 2000; Lee et al., 2003; Luczak & Ge, 1989). Uncertainty reflects the fact that artifacts of HF problems do not always occupy neat categories. Phenomena can be partially one thing and another (Walker, Salmon, Bedinger et al., 2017) and it is not always possible to have complete knowledge. It is for this reason that various fuzzy logic approaches have become popular (e.g., Masalonis & Parasuraman, 2003).

In complex sociotechnical systems, multiplicity can be observed as a large number of local interactions and multiple levels of system hierarchy. Multiplicity may also manifest itself as the presence of multiple potential causes for extant phenomena or the existence of multiple consequences of the eventualities afforded by these causes. Multiplicity can also be defined by multiplication of effects, where the effects of interactions within the system multiply and spread (e.g., Dekker et al., 2011). Many practical HF problems also comprise “multiple people” engaged in “complex team tasks” (e.g., Braarud, 2001; Stanton, 2014b). There are often “complex multiple tasks” involving “the control of a large number of interdependent process variables” (Sauer et al., 2000, p. 2044). Added to this are complex distributed teams and organizations, a heading under which these two strands (multiple tasks that are dealt with by multiple people) are brought together, often as a result of networked technology (e.g., Rogalski & Samurçay, 1993; Stanton et al., 2010). In essence, then, multiplicity may be recognized as the multiplicity of a systemic “feature” which contributes to the overall complexity of the system.

### Fuzzy Aggregation

The BBM uses fuzzy logic to pool a range of user inputs in order to position HF “problems” within a multidimensional “problem space” bounded by three axes: dynamism, uncertainty, and multiplicity. The same approach is used to position HF methods within a “method space.” . The two are blended into an “HF utility space” in which the match between method and problem can be assessed. This overall approach is analogous to one described by Smith (2006) and Alberts and Hayes (2006) and developed further by Walker, Stanton, Revell, et al. (2009) and Walker, Stanton, Salmon, et al. (2009).

Fuzzy logic is an approach used to compute degrees of truth rather than the strictly dichotomous true or false of Boolean binary logic (Zadeh, 1965). This is helpful for a problem like method selection in which true = 1 and false = 0 are difficult to know and thus too extreme (Enderton & Enderton, 2001). Fuzzy logic, therefore, includes the various states of truth in between 0 and 1 so that, for example, the result of a comparison between two things may not be true or false but “0.7” of true and, likewise, “0.3 of false” (Parasuraman et al., 2000). The capability to represent and account for various states of truth makes fuzzy logic a powerful tool for providing analytical insight to problems where variables cannot be measured binarily. Fuzzy logic is thus well suited to HF analysis where, often, the analyst is attempting to define, measure, and analyze dynamic units and interactions. As such, the approach has been used and discussed extensively in previous HF research (Karwowski & Mital, 2014; Konstandinidou et al., 2006; Li et al., 2010; Masalonis & Parasuraman, 2003; Parasuraman et al., 2000).

The three axes of the “problem” and “method spaces” used in the BBM represent the complexity attributes, with complexity increasing as “truth” scores increase from 0 to 1. A system or method’s complexity can be inferred from its coordinates in the problem and method spaces, respectively. The further out the system and/or method is along these coordinates, the more complex it is. The extent of closeness between “problem” and “method” is then assessed in the “utility space.” The distance between “problem” and “method” in the utility space provides a prediction of the level of PE for the method against the complexity exhibited by the problem. The shorter the distance between the method and the problem within the utility space, the more likely it is that the method will yield richer insights than methods with greater distance from the given problem. Whilst analysis of the utility space provides only a “prediction” of PE, the value offered to the practitioner is a measured approximation of the appropriateness of methods for tackling the given problem with the greatest efficiency.

The BBM process is described in detail below.

## Stage 1: Define The HF Problem Space

Based on a review and synthesis of contemporary accident causation models, Grant et al. (2018) identified a core set of “systems thinking tenets.” According to Grant et al. (2018), the 15 tenets represent the conditions and characteristics of work systems which, depending on their status, contribute to either unsafe or safe performance. The systems thinking tenets are presented in Table 2.

**Table 2:** Grant et al.’s Systems Thinking Tenets

Tenet	Description
Vertical integration	Mechanisms which allows decisions and actions at higher levels of a system to propagate down and influence behavior, and feedback regarding behavior to flow back up the hierarchy to influence decision making
Constraints	Aspects of the system which impose limits or influences on the behavior of system components
Normal performance	The manner in which activities are actually performed, regardless of standard operating procedures and formal rules
Performance variability	The natural variability in how tasks are performed
Emergence	Emergent behaviors and properties that arise as a result of interactions between system components
Functional dependencies	Necessary relationships between system components
Coupling	The relationship and dependencies that exist between system components
Nonlinear interactions	The nonlinearity of interactions which means minor tasks and components can interact to create unpredictable and significant outcomes
Linear interactions	Direct and predictable cause and effect relationships between system components
Feedback loops	Feedback mechanisms that enable communication between system components
Modularity	Subsystems and components that interact but are designed and operate independently of each other
Sensitive dependence on initial conditions	Original system conditions that influence system behavior
Decrementalism	The process of making minor modifications to system components that gradually create a significant change with safety risks
Unruly technologies	Unforeseen and unpredictable behaviors of new technologies that are introduced into the system
Contribution of the protective structure	The formal and organized structure that is intended to protect and optimize system safety but instead competes for resources with negative effects

Grant et al.’s (2018) system thinking tenets can be deployed in order to arrive at fuzzy truth values for the three overarching attributes of system complexity. Twelve system tenets provide fuzzy inputs to the HF problem space, with four tenets per attribute averaged to give final fuzzy scores for uncertainty, multiplicity, and dynamism. This provides a much more complete way of diagnosing system complexity rather than simply trying to judge complexity against broad “headline” attributes.

A worked example is provided in Table 3 below. It shows the ratings given to the system tenets based on a nuclear decommissioning case study. The full case study example is presented in Walker et al. (2014). It describes the decommissioning of a 2 million liter cartridge cooling pond (CCP). This involved retrieval, decontamination, and safe disposal of CCP apparatus, followed by removal of sludge, which had built up on surfaces within the pond, and then eventual draining of the pond itself. Before this stage was reached, however, two high-dose rate items had to be removed. It was vital, therefore, that all required measures were taken to protect operators participating in these tasks. From this arose the requirement to select the most appropriate HF method with which to analyze the task and identify all possible risks.

**Table 3:** System Tenets Grouped Under the Three Complexity Attributes of Dynamism, Uncertainty, and Multiplicity. Scores and Supporting Narrative Refer to a Nuclear Decommissioning Scenario Described in Walker et al. (2014)

Dynamism		
System Tenet	Score	Decision Log
*Emergence*—To what extent can an outcome or a property that is a result of the interactions between components in the system not be fully explained by examining the components alone?	0.5	Interactions between components may produce emergent system properties and outcomes that cannot be explained by examining components alone. In this problem, the potential for the interaction between various materials and isotopes may create new unforeseen hazards. Although comprehensive enabling studies would be undertaken prior to this task, the potential dynamism of the components and their interactions means a degree of emergence remains. Therefore, this tenet scores in the middle of the scale
*Decrementalization—*To what extent can small changes in normal performance gradually result in large changes?	0.8	This problem was felt to be prone to decrementalization, as small variances in normal performance (e.g., deviation from written procedures to speed up subtasks), which over time become ingrained, can be very common within a time-sensitive system
*Performance variability—*To what extent do systems and components change performance and behavior to meet the conditions in the world and environment in which the system must operate?	0.5	Different isotopes present different hazards and impose various performance demands for safe handling and disposal (e.g., various tools, levels of shielding). The properties of different isotopes, seen here as system components, introduce performance variability. The problem was judged to score at the midpoint for this tenet as the strictly controlled nature of nuclear decommissioning demands full knowledge of the inherent hazards of various isotopes and therefore, the performance(s) needed to safely handle and dispose of them are well defined
*Contribution of the protective structure—*To what extent does the protective structure of system control that is intended to optimize the system instead do the opposite? Does the protective structure inhibit performance variability? Does it introduce or impose new tasks that do not contribute to the goal? Are unnecessary controls introduced or imposed?	0.9	The contribution of the protective structure having a negative effect on system safety would likely manifest in the form of regulators either proposing or enforcing working methods/demands upon the system which, in reality, exacerbate working constraints and lead to conflicting goals within the organization. High-hazard scenarios under strict regulation are likely to produce high scores for this tenet
**Fuzzy Input (Average)**	**0.68**	
**Uncertainty System Tenet**	**Score**	**Decision Log**
*Unruly technology—*To what extent does technology introduce and sustain uncertainties about how and when things may fail?	0.2	The technology used to safely retrieve material is not likely to be novel and/or complex and operators will be suitably qualified and experienced to operate it. Unruliness of technology during these tasks cannot be fully discounted, however
*Sensitivity to initial conditions—*To what extent can seemingly negligible changes in initial conditions affect overall system behavior?	0.5	The initial conditions of this problem may be thought to be well understood; however, small changes to them or any errors or uncertainty in their determining can produce unpredictable system behavior (e.g., an error in contamination calculations can make the system more hazardous than expected or lead to different functional task requirements as a result of being over annual dose limit)
*Nonlinear interactions*—To what extent can/do interactions and relationships between components produce unpredictable outcomes? For example, seemingly inconsequential interactions have large effects on system behavior	0.5	Although linear interactions between system components are likely to be well understood, the potential for nonlinear interactions to compromise system safety still exists
*Inadequacy of feedback loops—*To what extent is the communication structure and information flow to evaluate control requirements of hazardous processes inadequate?	0.3	This problem is judged to have an adequate communication structure and information flow as prior to any tasks, there will likely be detailed enabling studies, extensive operator training, task trials, and lessons learned analyses. Therefore, well-defined communication structures and information flow are expected to exist
**Fuzzy Input (Average**)	**0.38**	
**Multiplicity System Tenet**	**Score**	**Decision Log**
*Modularity—*To what extent does normal performance depend on the interaction of an organization of subsystems and components which are designed and operated largely independently of each other?	0.7	Different actions from multiple actors and subsystems distributed across various levels and locations within the wider system are required to achieve a common goal. This problem is judged to present at a high level of modularity
*Vertical integration—*To what extent do multiple interactions between levels in the system hierarchy degrade communication/actions/decisions within the system?	0.8	The multitude of studies undertaken at different levels of the system hierarchy (e.g., initial isotope surveying, shielding required, tools and equipment required, operator competency, procedure writing, safe work instructions) must all be compatible to ensure system safety. The multiple interaction levels between these studies and documents could create competing priorities and conflicting information, severely degrading system safety. Therefore, the nature of vertical integration in this problem is likely to be very high
*Constraints—*What is the degree of multiplicity of system constraints which cause or influence limitations in the behaviors available to components within a system?	0.8	The multitude of constraints imposed on the problem by the wider system (e.g., expected dose rates, acceptable dose rates, time-sensitive subgoals) significantly adds to the multiplicity judged to exist throughout this problem
*Functional dependencies—*To what extent does the system exhibit multiple dependencies between system components that are not wanted or expected? For example, loss of communications due to poor signal	0.9	This problem is prone to various functional dependencies as system safety is dependent on accuracy of dose rate calculations, adequate shielding, clear communication channels, supply of appropriate tools, and a variety of other critical system components
**Fuzzy Input (Average**)	**0.8**	

The BBM is designed for practical use to deliver benefits in terms of an output (a set of methods to match an extant problem) and a process (to provide benefit to practitioners by helping them to think and reason about complexity). Table 3 represents a template others can use in their own work. It contains the relevant system tenet, space for the fuzzy logic scores to be entered, and a decision log to explain the rationale. The ratings shown in Table 3 are an example derived from a HF/system safety specialist with experience in safety critical domains (nuclear and rail). It is recommended that this rating activity is conducted as a teamwork exercise involving a diverse and multidisciplinary team including but not limited to HF professionals, system design experts, risk managers, and system operators. The goal is to enable teams to think collectively and to build consensus about the complexity of the system within which their HF problem is nested.

**Figure 3:**
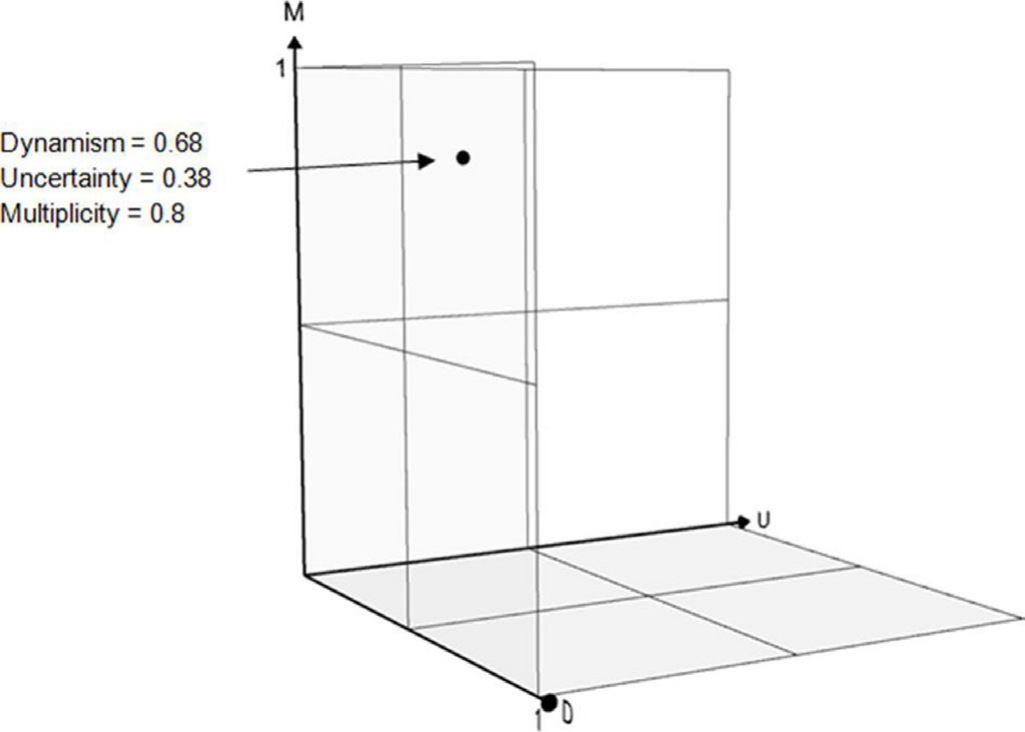
HF problems space consisting of intersecting *x* (uncertainty), *y* (multiplicity), and *z* (dynamism) attribute axes. Plotted into the space is the cartridge cooling pond case study under analysis with the process of fuzzy aggregation providing the coordinates.

The aggregate fuzzy values for each complexity attribute are plotted on the 3D graph shown in Figure 3, the so-called HF problem space. The case study system, rated by the subject-matter expert according to the fuzzy logic rubric, has been plotted. It shows where the nuclear decommissioning case study is felt to reside in the HF problem space (0.68 for dynamism; 0.38 for uncertainty; 0.8 for multiplicity). It indicates a high amount of complexity.

## Stage 2: Define The HF Method Space

Having fixed the HF problem into the problem space, and in doing so characterized its complexity, the next step is to do the same for HF methods. These are selected from Stanton et al. (2013) as representing those methods which are available, applicable, original, and not domain specific. A hundred and seven methods meet these criteria. From this text, and in view of the nuclear case study aims, only methods which can be used predictively to identify hazards have been selected for analysis via the BBM. As such, methods which are designed to be used retrospectively (e.g., AcciMap) have been omitted from the present analysis. These remaining 66 methods are shown in Table 4.

**Table 4:** Selection of Predictive HF Methods Broadly Applicable to the Nuclear Decommissioning Case Study and Suitable for Inclusion in the HF Problem Space

Method	Acronym	Key Reference
Data Collection Methods		
Interviews	I	Various
Questionnaires	Q	Various
Observation	O	Baber and Stanton (1996)
Mouse tracking	MT	Various
Task Analysis Methods		
Hierarchal Task Analysis	HTA	Annett et al. (1971)
Goals Operators Methods and Selection Rules	GOMS	Card, Moran, Newell et al. (1983)
Verbal Protocol Analysis	VPA	Walker (2004)
Task Decomposition	TD	Kirwan and Ainsworth (1992)
Subgoal Templates	SGT	Ormerod and Shepherd (2003)
Tabular Task Analysis	TTA	Kirwan (1994)
Cognitive Task Methods		
Applied Cognitive Task Analysis	ACTA	Militello and Hutton (2000)
Cognitive Walkthrough	CW	Polson et al. (1992)
Critical Decision Method	CDM	Klein and Armstrong (2004)
Concurrent Observer Narrative Technique	CONT	McIlroy and Stanton (2011)
Collegial Verbalisation	CV	Erlandsson and Jansson (2007)
Objective-Orientated Cognitive Task Analysis and Design	OOCTAD	Wei and Salvendy (2006)
Process Charting Methods		
Operational Sequence Diagram	OSD	Kirwan and Ainsworth (1992)
Event Tree Analysis	ETA	Kirwan and Ainsworth (1992)
Decision Action Diagram	DAD	Kirwan and Ainsworth (1992)
Fault Tree	FT	Kirwan and Ainsworth (1992)
Human Error Prediction Methods		
Systematic Human Error Reduction & Prediction Approach	SHERPA	Embrey (1986)
Human Error Template	HET	Young et al. (2003)
Technique for Retrospective and Predictive Analysis of Cognitive Errors	TRACEr	Shorrock and Kirwan (2002)
Task Analysis for Error Identification	TAFEI	Stanton and Baber (1996)
Human Error–Hazard and Operability study	HE-HAZOP	Kletz (1974); Swann and Preston (1995)
Technique for Human Error Assessment	THEA	Pocock et al. (2001)
Human Error Identification in Systems Tool	HEIST	Kirwan (1994)
Human Error and Recovery Assessment	HERA	Kirwan (1998a, 1998b)
System for Predictive Error Analysis and Reduction	SPEAR	Center for Chemical Process Safety
Human Error Assessment and Reduction Technique	HEART	Williams (1986)
Cognitive Reliability and Error Analysis Method	CREAM	Hollnagel (1998)
Situational Awareness Methods		
Situational Awareness Requirements Analysis	SARA	Endsley (2001)
Situation Awareness Global Assessment Technique	SAGAT	Endsley (1995)
Situation Present Assessment Method	SPAM	Durso et al. (1998)
Situation Awareness Rating Technique	SART	Taylor (1990)
Situational Awareness Subjective Workload Dominance	SA-SWORD	Vidulich and Hughes (1991)
Mental Workload Methods		
National Aeronautics and Space Administration Task Load Index	NASA TLX	Hart and Staveland (1988)
Modified Cooper Harper Scale	MCH	Cooper and Harper (1969)
Subjective Workload Assessment Technique	SWAT	Reid and Nygren (1988)
Projective Subjective Workload Assessment Technique	Pro-SWAT	Reid and Nygren (1988)
DRA Workload Scales	DRAWS	Jordan et al. (1995)
Malvern Capacity Estimate Technique	MACE	Goillau and Kelly (1996)
Workload Profile Technique	WPT	Tsang and Velazquez (1996)
Bedford Scale	BS	Roscoe and Ellis (1990)
Instantaneous Self-Assessment	ISA	Kirwan et al. (1997)
Cognitive Task Load Analysis	CTLA	Neerincx (2003)
Projective Subjective Workload Dominance Technique	Pro-SWORD	Vidulich and Hughes (1991)
Mental Workload Index	MWLI	Pretorius and Cilliers (2007)
Team Analysis		
Communications Usage Diagram	CUD	Watts and Monk (2000)
Coordination Demand Analysis	CDA	Burke (2004)
Decision Requirements Exercise	DRX	Klinger and Hahn (2004)
Groupware Task Analysis	GTA	Van Welie and van der Veer (2003)
Hierarchal Task Analysis for Teams	HTA(*T*)	Annett (1997)
Teams Cognitive Task Analysis	TCTA	Klein (2000)
Social Network Analysis	SNA	Driskell and Mullen (2005)
Questionnaires for Distributed Assessment for Team Mutual Awareness	QDATMA	MacMillan et al. (2004)
Team Workload Assessment	TWA	Bowers and Jentsch (2005)
Task and Training Requirements Analysis Methodology	TTRAM	Swezey et al. (2000)
Cockpit Management Attitudes Questionnaire	CMAQ	Helmreich (1984)
Targeted Acceptable Responses to Generated Events or Tasks	TARGETs	Fowlkes et al. (1994)
Time Performance		
Multi-Modal Critical Path Analysis	MMCPA	Baber and Mellor (2001)
Timeline Analysis	TimA	Kirwan and Ainsworth (1992)
Systems Methods		
Cognitive Work Analysis	CWA	Rasmussen et al. (1994)
Event Analysis of Systemic Teamwork	EAST	Stanton et al. (2013)
Functional Resonance Analysis Method	FRAM	Hollnagel (2004)
Systems Theoretic Accident Modeling and Processes model	STAMP	Leveson (2004)

Each method is then subject to the same rating system used for defining the HF problem space. The method rating exercise involves rating the methods for their capability in tackling each tenet on 11-point scales of 0 to 1 (0, 0.1, 0.2, …) to produce overall fuzzy ratings relating to their capability of tackling varying degrees of dynamism, uncertainty, and multiplicity (see Appendix 1 for the methods rating questionnaire). For this particular case study, the process was performed by the author team and the SME. Whilst the small sample size limits the validity of the present reported ratings, they are not intended to be fixed method ratings at this stage. Rather, they represent an exercise in running the BBM process and building a consensus around the particular case study problem.

A further consideration is that the questionnaire does not offer strict criteria from which the method ratings are derived. The limitation of this is the subjectivity of the method ratings. This may be addressed by two means: one in the short term and one longer term. First, methods rated by an analyst team performing ratings can be subject to a basic sensitivity analysis through the application of uncertainty bounds. The supporting calculation spreadsheet enables these uncertainty bounds to be applied so that in future final ratings can represent the midpoint of a specified range. This function allows users to specify an uncertainty range for method ratings, where for example a range of 0.1 creates upper and lower bounds of *n* ± 0.05. The supplementary spreadsheet accounts for the upper and lower bounds for each method in the final utility ratings, where three eventual ratings are generated; the midpoint, the lower uncertainty bounds, and the upper uncertainty bounds. Whilst application of uncertainty bounds would not strengthen the statistical validity of the method ratings process, it would somewhat mitigate the impact of subjective method ratings on calculated PE values.

The longer term measure would be to validate the method ratings over time. The materials used to generate these ratings are made publicly available for use and reuse; the initial ratings performed by the authors could be used as a starting point from which other practitioner and research teams perform their own ratings. The objective of this would be the eventual generation of a database of ratings which could be aggregated to form a “finalized” set of method ratings. Furthermore, if enough data were collected, statistically derived uncertainty bounds could be applied to method ratings to strengthen their validity.

In the fullness of time it is hoped that wider discipline consensus can be reached around method capability, but that is by no means an essential precursor to using the BBM approach. As stated previously, the BBM “process” is as important as the “output.” All that being said, rating the methods and placing them in the method space reveal some important findings which relate well to wider debates about systems methods in the literature. The full ratings are shown in Table 5. No uncertainty bounds have been applied to these ratings; however as noted, the supplementary spreadsheet includes an uncertainty bounds function which allows analysts to specify an arbitrary uncertainty range for the method attribute ratings (i.e., dynamism, uncertainty, and multiplicity). This generates a series of corresponding upper and lower values for the method ratings which carry through the overall upper and lower PE ratings.

**Table 5:** Table of Fuzzy Logic Ratings for Each of the Candidate Methods and the Cartesian Distance Between the Method and the Case Study Problem. Methods Yielding the Highest Measure of Predictive Efficiency in Each Category for Tackling the Case Study Problem Are Listed in Bold

Method	Ratings (0–1)																Predictive Efficiency
	Dynamism					Uncertainty					Multiplicity						
**Data Collection**	Emer	Dec	PV	CPS	**Ave**	UT	StIC	NLI	FL	**Ave**	Mod	VI	Cons	FD	**Ave**	**Score** (***D*,*U*,*M***)	**Cartesian Distance** (***d***)
**Interviews**	0.2	0.4	0.4	0.1	**0.28**	0.1	0.1	0.4	0.3	**0.23**	0.1	0.5	0.3	0.2	**0.28**	**0.28, 0.23, 0.28**	**0.68**
Questionnaires	0.1	0	0.1	0.2	**0.1**	0.1	0	0	0.1	**0.05**	0.3	0.3	0.2	0.2	**0.25**	**0.1, 0.05, 0.25**	0.79
Observation	0.2	0.1	0.4	0.1	**0.2**	0.2	0.1	0.2	0.2	**0.18**	0.1	0.3	0.2	0.2	**0.2**	**0.2, 0.18, 0.2**	0.86
Mouse tracking	0.1	0	0.3	0.1	**0.13**	0.2	0	0.1	0.1	**0.1**	0	0	0.2	0.1	**0.08**	**0.13, 0.1, 0.08**	0.95
**Task Analysis**	Emer	Dec	PV	CPS	**Ave**	UT	StIC	NLI	FL	**Ave**	Mod	VI	Cons	FD	**Ave**	**Score** (***D*,*U*,*M***)	**Cartesian Distance** (***d***)
HTA	0.1	0.1	0.2	0.1	**0.13**	0.3	0.2	0.4	0.2	**0.28**	0.2	0.2	0.3	0.1	**0.2**	**0.13, 0.28, 0.2**	0.82
**GOMS**	0.2	0.1	0.5	0	**0.2**	0.6	0.1	0.3	0.2	**0.3**	0.3	0.1	0.3	0.3	**0.25**	**0.2, 0.3, 0.25**	**0.73**
VPA	0.3	0.2	0.3	0.1	**0.2**	0.1	0.1	0.2	0.1	**0.13**	0.1	0.2	0.4	0.3	**0.23**	**0.2, 0.13, 0.23**	0.75
TD	0.1	0.1	0.2	0.1	**0.13**	0.2	0.2	0.2	0.3	**0.23**	0.1	0.3	0.2	0.3	**0.23**	**0.13, 0.23, 0.23**	0.81
SGT	0.1	0.1	0.2	0.1	**0.13**	0.3	0.2	0.4	0.2	**0.28**	0.2	0.2	0.3	0.1	**0.2**	**0.13, 0.28, 0.2**	0.82
TTA	0.1	0.1	0.2	0.1	**0.13**	0.3	0.2	0.4	0.2	**0.28**	0.2	0.2	0.3	0.1	**0.2**	**0.13, 0.28, 0.2**	0.82
**Cognitive Task Analysis**	Emer	Dec	PV	CPS	**Ave**	UT	StIC	NLI	FL	**Ave**	Mod	VI	Cons	FD	**Ave**	**Score** (***D*,*U*,*M***)	**Cartesian Distance** (***d***)
**ACTA**	0.4	0.2	0.7	0.4	**0.43**	0.3	0.4	0.5	0.4	**0.4**	0.5	0.5	0.6	0.5	**0.53**	**0.43, 0.4, 0.53**	**0.37**
CW	0.1	0.2	0.3	0	**0.15**	0.6	0	0.1	0.1	**0.2**	0	0	0.1	0.1	**0.05**	**0.15, 0.2, 0.05**	0.93
CDM	0.5	0.3	0.6	0.1	**0.38**	0.2	0.3	0.4	0.4	**0.33**	0.5	0.6	0.6	0.4	**0.53**	**0.38, 0.33, 0.53**	0.41
CONT	0.4	0.2	0.3	0.1	**0.23**	0.1	0.1	0.4	0.2	**0.2**	0.1	0.2	0.4	0.4	**0.28**	**0.23, 0.2, 0.28**	0.70
CV	0.3	0.1	0.6	0.1	**0.28**	0.4	0.1	0.6	0.7	**0.45**	0.4	0.5	0.5	0.5	**0.48**	**0.28, 0.45, 0.48**	0.52
OOCTAD	0.3	0.1	0.5	0.1	**0.25**	0.5	0.2	0.4	0.5	**0.4**	0.2	0.2	0.4	0.3	**0.28**	**0.25, 0.4, 0.28**	0.68
**Process Charting**	Emer	Dec	PV	CPS	**Ave**	UT	SIC	NLI	FL	**Ave**	Mod	VI	Cons	FD	**Ave**	**Score** (***D*,*U*,*M***)	**Cartesian Distance** (***d***)
**OSD**	0.2	0.1	0.2	0.2	**0.18**	0.3	0.2	0.5	0.5	**0.45**	0.5	0.4	0.6	0.5	**0.48**	**0.18, 0.45, 0.48**	**0.58**
ETA	0.3	0.1	0.4	0.1	**0.2**	0.1	0.2	0.1	0.2	**0.15**	0.2	0.2	0.3	0.3	**0.25**	**0.2 0.15, 0.25**	0.75
DAD	0.1	0	0.1	0.1	**0.08**	0.1	0.2	0.1	0.3	**0.18**	0.3	0.2	0.2	0.2	**0.23**	**0.08, 0.18, 0.23**	0.85
FT	0.2	0.1	0.3	0.1	**0.18**	0.1	0.2	0.1	0.2	**0.15**	0.2	0.2	0.3	0.3	**0.25**	**0.18, 0.15, 0.25**	0.78
**Human Error ID**	Emer	Dec	PV	CPS	**Ave**	UT	StIC	NLI	FL	**Ave**	Mod	VI	Cons	FD	**Ave**	**Score** (***D*,*U*,*M***)	**Cartesian Distance** (***d***)
SHERPA	0.2	0.1	0.2	0.1	**0.15**	0.1	0.1	0.1	0.2	**0.13**	0.2	0.1	0.2	0.1	**0.1**	**0.15, 0.13, 0.1**	0.87
HET	0.2	0.1	0.1	0.1	**0.13**	0.2	0.1	0.1	0.1	**0.13**	0.2	0.2	0.2	0.3	**0.15**	**0.13, 0.13, 0.15**	0.83
TRACEr	0.3	0.2	0.4	0.2	**0.23**	0.4	0.2	0.4	0.6	**0.4**	0.3	0.2	0.3	0.4	**0.3**	**0.23, 0.4, 0.3**	0.64
TAFEI	0.3	0.3	0.2	0.1	**0.23**	0.5	0.3	0.7	0.7	**0.55**	0.5	0.6	0.3	0.4	**0.45**	**0.23, 0.55, 0.45**	0.60
HE-HAZOP	0.3	0.2	0.4	0.2	**0.28**	0.4	0.3	0.3	0.3	**0.33**	0.2	0.4	0.5	0.4	**0.38**	**0.28, 0.33, 0.38**	0.59
THEA	0.2	0.1	0.3	0.1	**0.18**	0.7	0.2	0.4	0.4	**0.43**	0.2	0.1	0.4	0.2	**0.23**	**0.18, 0.43, 0.23**	0.76
HEIST	0.6	0.2	0.5	0.1	**0.35**	0.4	0.4	0.6	0.6	**0.5**	0.3	0.3	0.4	0.4	**0.35**	**0.35, 0.5, 0.35**	0.57
HERA	0.6	0.3	0.5	0.3	**0.43**	0.5	0.4	0.6	0.7	**0.55**	0.4	0.3	0.4	0.4	**0.38**	**0.43, 0.55, 0.38**	0.52
SPEAR	0.3	0.2	0.3	0.2	**0.25**	0.2	0.1	0.2	0.3	**0.2**	0.3	0.2	0.3	0.2	**0.25**	**0.25, 0.2, 0.25**	0.72
HEART	0.1	0.1	0.3	0.1	**0.15**	0.4	0.2	0.2	0.2	**0.25**	0.2	0.2	0.3	0.2	**0.23**	**0.15, 0.25, 0.23**	0.79
**CREAM**	0.5	0.6	0.7	0.5	**0.58**	0.7	0.4	0.6	0.7	**0.6**	0.5	0.7	0.6	0.7	**0.63**	**0.58, 0.6, 0.63**	**0.30**
**Situation Awareness**	Emer	Dec	PV	CPS	**Ave**	UT	StIC	NLI	FL	**Ave**	Mod	VI	Cons	FD	**Ave**	**Score** (***D*,*U*,*M***)	**Cartesian Distance** (***d***)
SARA	0.3	0.3	0.4	0.4	**0.35**	0.3	0.2	0.3	0.4	**0.3**	0.2	0.2	0.4	0.3	**0.28**	**0.35, 0.3, 0.28**	0.62
SAGAT	0.2	0.3	0.5	0.3	**0.33**	0.5	0.4	0.3	0.4	**0.4**	0.2	0.2	0.4	0.3	**0.28**	**0.33, 0.4, 0.28**	0.63
SPAM	0.1	0.2	0.2	0.1	**0.15**	0.3	0.2	0.2	0.3	**0.25**	0.2	0.1	0.3	0.3	**0.23**	**0.15, 0.25, 0.23**	0.79
**SART**	0.5	0.3	0.6	0.3	**0.43**	0.2	0.7	0.7	0.6	**0.55**	0.4	0.6	0.6	0.5	**0.53**	**0.43, 0.55, 0.53**	**0.41**
SA-SWORD	0.3	0.2	0.4	0.1	**0.25**	0.3	0.2	0.3	0.3	**0.28**	0.2	0.2	0.3	0.2	**0.23**	**0.25, 0.28, 0.23**	0.72
**Mental Workload**	Emer	Dec	PV	CPS	**Ave**	UT	StIC	NLI	FL	**Ave**	Mod	VI	Cons	FD	**Ave**	**Score** (***D*,*U*,*M***)	**Cartesian Distance** (***d***)
NASA TLX	0.1	0	0.1	0.2	**0.1**	0.2	0.1	0.1	0.1	**0.13**	0.1	0.2	0.2	0.1	**0.15**	**0.1, 0.13, 0.15**	0.90
MCH	0.1	0	0.2	0	**0.08**	0.3	0.1	0.2	0.1	**0.18**	0.1	0.1	0.1	0.1	**0.1**	**0.08, 0.18, 0.1**	0.94
SWAT	0.1	0	0.1	0	**0.05**	0.3	0.1	0.1	0.1	**0.15**	0.1	0.2	0.2	0.1	**0.15**	**0.05, 0.15, 0.15**	0.93
Pro-SWAT	0.1	0	0.1	0	**0.05**	0.3	0.1	0.1	0.1	**0.15**	0.1	0.2	0.2	0.1	**0.15**	**0.05, 0.15, 0.15**	0.93
DRA	0.1	0	0.2	0	**0.08**	0.2	0.1	0.1	0.2	**0.15**	0.1	0.2	0.2	0.1	**0.15**	**0.08, 0.15, 0.15**	0.91
MACE	0.1	0	0.2	0	**0.08**	0.2	0.1	0.1	0	**0.1**	0.1	0.1	0.2	0	**0.1**	**0.08, 0.1, 0.1**	0.96
WPT	0.2	0	0.3	0.1	**0.15**	0.3	0.2	0.2	0.2	**0.23**	0.1	0.1	0.2	0.2	**0.15**	**0.15, 0.23, 0.15**	0.85
BS	0.1	0	0.1	0	**0.05**	0.1	0	0.1	0	**0.05**	0	0	0.1	0	**0.03**	**0.05, 0.05, 0.03**	1.05
ISA	0.2	0	0.2	0.1	**0.13**	0.2	0.2	0.1	0.1	**0.15**	0.1	0.1	0.1	0.1	**0.1**	**0.13, 0.15, 0.1**	0.92
**CTLA**	0.3	0.1	0.3	0.1	**0.2**	0.3	0.2	0.2	0.2	**0.23**	0.2	0.1	0.2	0.3	**0.2**	**0.2, 0.23, 0.2**	**0.78**
Pro-Sword	0.1	0	0.1	0	**0.05**	0.2	0.1	0.1	0.1	**0.13**	0.1	0.1	0.2	0.1	**0.13**	**0.05, 0.13, 0.13**	0.95
MWLI	0.2	0.1	0.2	0.2	**0.18**	0.3	0.2	0.1	0.3	**0.23**	0.2	0.1	0.2	0.2	**0.18**	**0.18, 0.23, 0.18**	0.81
**Team Analysis**	Emer	Dec	PV	CPS	**Ave**	UT	StIC	NLI	FL	**Ave**	Mod	VI	Cons	FD	**Ave**	**Score** (***D*,*U*,*M***)	**Cartesian Distance** (***d***)
CUD	0.3	0.2	0.4	0.2	**0.23**	0.4	0.2	0.3	0.4	**0.33**	0.4	0.6	0.4	0.3	**0.43**	**0.23, 0.33, 0.43**	0.55
CDA	0.3	0.3	0.4	0.2	**0.3**	0.4	0.3	0.3	0.4	**0.35**	0.4	0.6	0.4	0.3	**0.43**	**0.3, 0.35, 0.43**	0.53
DRX	0.2	0.2	0.4	0.2	**0.25**	0.4	0.2	0.3	0.4	**0.33**	0.3	0.6	0.4	0.3	**0.4**	**0.25, 0.33, 0.4**	0.59
GTA	0.5	0.4	0.5	0.5	**0.48**	0.6	0.3	0.5	0.7	**0.53**	0.3	0.6	0.6	0.4	**0.48**	**0.48, 0.53, 0.48**	0.41
HTA(T)	0.1	0.1	0.2	0.1	**0.13**	0.3	0.2	0.4	0.2	**0.28**	0.2	0.2	0.3	0.1	**0.2**	**0.13, 0.28, 0.2**	0.82
CTA(T)	0.4	0.3	0.7	0.4	**0.45**	0.4	0.4	0.6	0.8	**0.65**	0.4	0.7	0.5	0.5	**0.53**	**0.45, 0.65, 0.53**	0.40
**SNA**	0.5	0.4	0.6	0.4	**0.48**	0.4	0.3	0.5	0.6	**0.45**	0.6	0.7	0.6	0.3	**0.55**	**0.48, 0.45, 0.55**	**0.33**
QDATMA	0.3	0.2	0.4	0.3	**0.3**	0.3	0.2	0.3	0.2	**0.25**	0.4	0.3	0.5	0.3	**0.38**	**0.3, 0.25, 0.38**	0.58
TWA	0.3	0.2	0.4	0.3	**0.3**	0.3	0.2	0.3	0.2	**0.25**	0.4	0.3	0.5	0.3	**0.38**	**0.3, 0.25, 0.38**	0.58
TTRAM	0.2	0.5	0.6	0.2	**0.38**	0.5	0.3	0.3	0.3	**0.35**	0.4	0.3	0.5	0.3	**0.38**	**0.38, 0.35, 0.38**	0.52
CMAQ	0.3	0.6	0.5	0.5	**0.48**	0.2	0.4	0.4	0.5	**0.38**	0.3	0.5	0.5	0.3	**0.4**	**0.48, 0.38, 0.4**	0.45
TARGETs	0.3	0.5	0.5	0.3	**0.4**	0.3	0.3	0.4	0.4	**0.35**	0.3	0.5	0.4	0.3	**0.38**	**0.4, 0.35, 0.38**	0.51
**Time Performance**	Emer	Dec	PV	CPS	**Ave**	UT	StIC	NLI	FL	**Ave**	Mod	VI	Cons	FD	**Ave**	**Score** (***D*,*U*,*M***)	**Cartesian Distance** (***d***)
**CPA**	0.5	0.3	0.5	0.4	**0.43**	0.2	0.4	0.3	0.6	**0.38**	0.5	0.5	0.7	0.6	**0.58**	**0.43, 0.38, 0.58**	**0.34**
TimA	0.5	0.4	0.6	0.4	**0.48**	0.3	0.4	0.4	0.6	**0.43**	0.5	0.6	0.5	0.4	**0.4**	**0.48, 0.43, 0.4**	0.36
**Systems Methods**	**Emer**	**Dec**	**PV**	**CPS**	**Ave**	**UT**	**StIC**	**NLI**	**FL**	**Ave**	**Mod**	**VI**	**Cons**	**FD**	**Ave**	**Score** (***D*,*U*,*M***)	**Cartesian Distance** (***d***)
FRAM	0.6	0.7	0.9	0.9	**0.78**	0.5	0.5	0.8	0.8	**0.65**	0.8	0.7	0.9	1	**0.85**	**0.78, 0.65, 0.85**	0.30
EAST	0.8	0.5	0.8	0.5	**0.65**	0.8	0.6	0.9	0.9	**0.8**	0.6	0.7	0.7	0.7	**0.68**	**0.65, 0.8, 0.68**	0.44
**CWA**	0.6	0.7	0.8	0.8	**0.73**	0.4	0.4	0.8	0.8	**0.6**	0.9	0.8	1	0.8	**0.88**	**0.73, 0.6, 0.88**	**0.24**
STAMP	0.5	0.8	0.9	1	**0.80**	0.7	0.4	0.6	0.9	**0.65**	0.5	0.9	1	0.7	**0.83**	**0.8, 0.65, 0.83**	**0.30**

**Figure 4:**
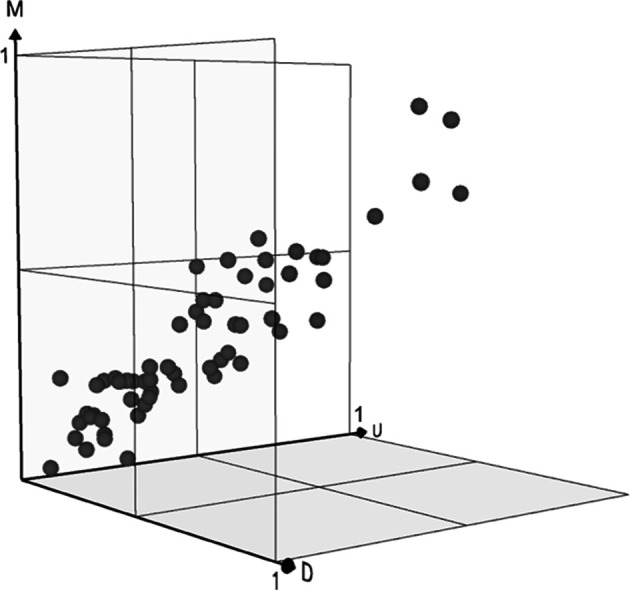
Distribution of methods across the method space by octant. Coordinates for octants shown in Table 6. Axes are defined as *x* (uncertainty), *y* (multiplicity), and *z* (dynamism).

The [*d*,*u*,*m*] data points shown in Table 5 and Figure 4 serve as the upper bound of method capability. It is generally assumed that a method could (potentially) operate anywhere from [0,0,0] up to its maximum value, albeit with suboptimal PE. For example, the high scoring CWA method “could” be applied to problems with low complexity scores, but the risk is of “overkill” in relation to the level of insight delivered. The BBM makes these trade-offs explicit.

Of course, the inherent subjectivity of this simple demonstration means it is sensible to widen the frame and to consider method matching in a broader sense. One way to achieve this is to divide the utility space into eight octants, each one representing high or low multiplicity, dynamism, or uncertainty and a particular type of complexity. This shifts the perspective so that instead of defining a “point” in the “spaces” one is defining “regions.” This desensitizes the analysis endeavor to the vagaries of individual fuzzy method ratings and allows broader latitude in method selection. In other words, the approach allows practitioners to pick from a methods “pallet” rather than recommend just one method to the exclusion of all others.

The distribution of methods across octants is shown in Figure 4 and corresponding coordinates for each octant shown in Table 6. It becomes evident that 51 out of 66 methods (77%) fall within Octant 1, which is characterized by low dynamism, low uncertainty, and low multiplicity. According to the same ratings just 5 out of 66 methods (7%) fall within Octant 8 and the region of high dynamism, high uncertainty, and high multiplicity. Three regions of the method space (Octants 5, 6, and 7) are not serviced at all by the methods appropriate to this case study, at least with optimum PE.

**Table 6:** Distribution of the 66 Methods Across Method Space Octants

Octant	Method Space Coordinates *x* (Uncertainty), *y* (Dynamism), *z* (Multiplicity)	Total	~%
1	([0,0.5], [0,0.5], [0,0.5])	51	77
2	([0,0.5], [0,0.5], [0.5,1])	3	5
3	([0,0.5], [0.5,1], [0,0.5])	4	6
4	([0,0.5], [0.5,1], [0.5,1])	2	3
5	([0.5,1], [0,0.5], [0,0.5])	0	-
6	([0.5,1], [0,0.5], [0.5,1])	0	-
7	([0.5,1], [0.5,1], [0,0.5])	0	-
8	([0.5,1), [0.5,1], [0.5,1])	5	7

## Stage 3: Define The HF Utility Space

**Figure 5:**
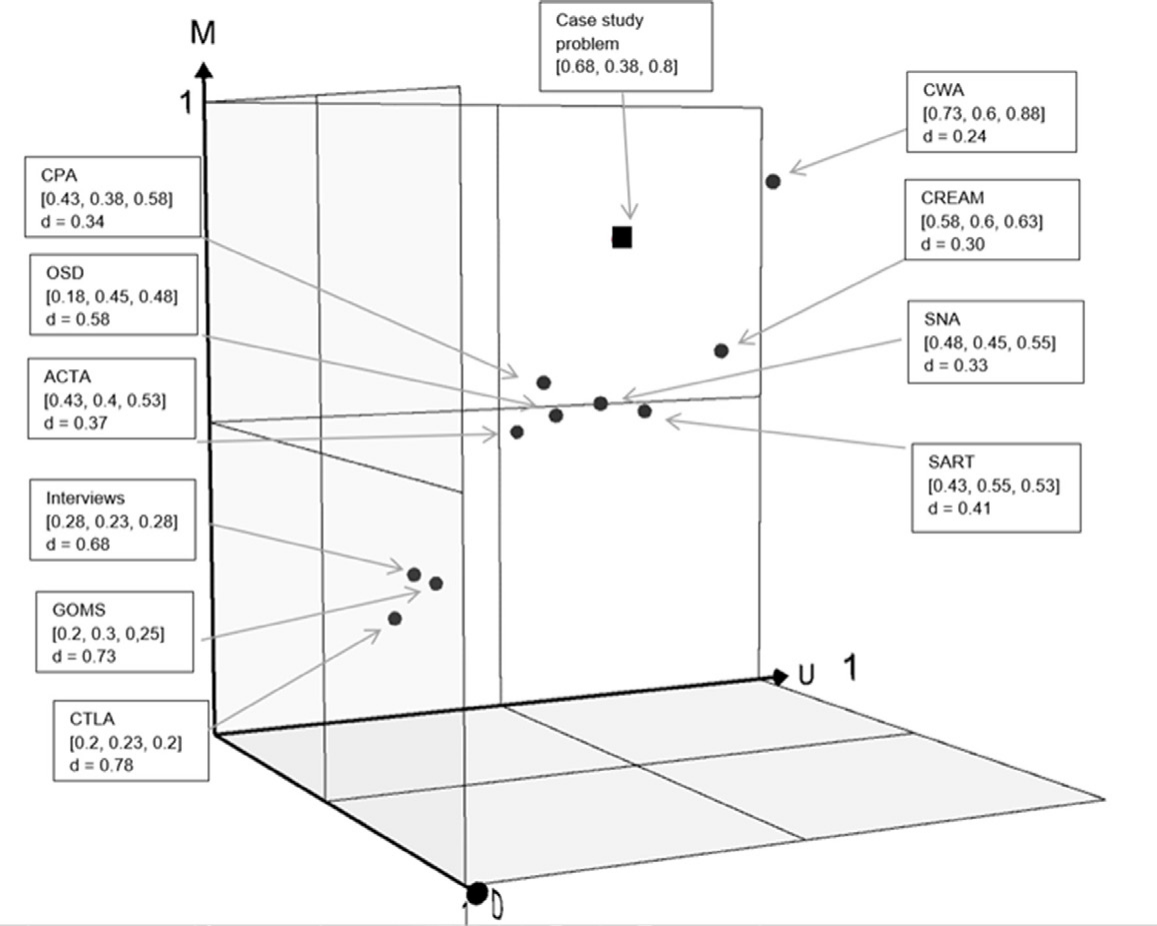
HF utility space consisting of intersecting *x* (uncertainty), *y* (multiplicity), and *z* (dynamism) attribute axes. Plotted into the space are methods with the lowest Cartesian distance from the “problem” for each of the 10 method categories.

HF problem and approach spaces have been created. The two “spaces” have identical axes—dynamism, uncertainty, and multiplicity—along which any HF problem (in this case a nuclear decommissioning case study) and any HF method (in this case 66 individual methods) are rated using a fuzzy aggregation process. This enables both the target HF problem and the target HF method(s) to be positioned in this space. Since the same system thinking tenets and metrics are used, the method space and problem space have identical scales. This internal consistency enables the final conceptual “space” to be created, the utility space. This is a space in which problems and methods coexist within consistent conceptual parameters (Figure 5). The goal is to ascertain which HF method is a conceptual match—on dynamism, uncertainty, and multiplicity—to the target HF problem. The Cartesian distance between the method(s) and the problem within the utility space enables this, and is calculated as follows:

$$d=\sqrt{(x_{2}-x_{1}{)}^{2}+y_{2}-y_{1}{)}^{2}+z_{2}-z_{1}{)}^{2}}$$

where

*d* = Cartesian distance within the utility space, *n*_2_ = the HF method and *n*_1_ = the HF problem.

As discussed, the calculated distance between HF problems and methods provides a theoretical prediction of PE. Using this formula, the shortest distance between the HF problem and its respective method is the one that is predicted to achieve the highest PE, in other words, which has the most favorable ratio between analysis effort and resultant insight.

For the case study at hand, the method with the lowest Cartesian distance within the utility space, and which therefore has the highest PE, is CWA (*d* = 0.24). To be abundantly clear, these are methods matched on PE grounds as being most appropriate to the specific case study presented above. It is not an absolute judgment on whether one method is better or worse than another. To be clear again, the specific methods required of a particular analysis depend on the question(s) being asked which, of course, are decided by the analyst/client. For example, if a situation awareness analysis method matched to the problem at hand is required then the SART meets the criteria (*d* = 0.41) compared to SPAM (*d* = 0.79); if an error identification method is sought then CREAM is a closer match (*d* = 0.30) than HEART (*d* = 0.79); and so on. The methods in this case have been positioned at the upper bounds of their complexity capabilities, but if the analysis warrants lower levels of PE then methods can move downwards (but not upwards). The final key point to reiterate is that whatever the subjectivity in the present case study, the trade-offs in method suitability are made explicit. The “process” of applying the BBM is as worthwhile as the “output,” and the full BBM materials are made available for wider consensus building going forward.

The authors have made their best effort to discuss limitations to the model as they arise; however, some further considerations must be brought to the reader’s attention. First, the authors acknowledge that whilst the use of PE as an analog for the utility of methods for addressing problems supports the goals of the BBM, its internal mechanism may seem at odds with the growing body of systems thinking and resilience engineering literature (Grant et al., 2018; Hollnagel et al., 2006; Leveson et al., 2006; Woods et al., 2017). This point needs to be clarified. In the present paper, PE is discussed in terms of “predicting outcomes” based on the amount of entropy in a system. The resilience engineering and systems thinking schools of thought fundamentally question the value of the mere prediction of outcomes. This is because little insight is provided into the potentially innumerable complex factors preceding the terminal event. Thus, matching methods to problems of increasing complexity based on their appropriateness for predicting outcomes seems inconsistent. This is not the intended reading of the BBM approach. Utilizing PE for the BBM is relevant to the selection of appropriate methodology only. For instance, the example included above shows CWA to be the method representing the strongest PE for the given problem. The intention of the BBM is not to advocate the use of CWA for merely predicting outcomes (e.g., human performance variability, human error potential, hazardous system states). Rather, CWA is recommended based on the appropriateness of its full analytical scope for coping with the complexity exhibited by the problem, where prediction or modeling of the aforementioned phenomena may be a subset of potentially numerous CWA findings. This distinction is important. The BBM may recommend the use of methods with far more sophistication and potential than those which solely focus on prediction of outcomes. Thus, the use of PE to underpin the BBM utility space does not reflect an inherent advocacy for the value of HF or accident causation methods focussing on prediction of outcomes only. As problems become more complex, the prediction of outcomes becomes less meaningful, and becomes less of a feature of methods the BBM recommends.

A final consideration is the impacts of the reduction of complexity into a three-dimensional space. Such an approach is not unusual. In complexity science similar multidimensional spaces, called “phase spaces,” are used to trace the relationship between discrete variables and date from physicist Willard Gibbs’ pioneering work at the turn of the century (Gibbs, 1902). Unlike physical systems, many of the variables of interest in HF, including those relevant to method selection, are not reducible to a set of fundamental equations. Methods such as functional holography (Baruchi et al., 2006), for example, use principal components analysis (PCA) instead to collapse *N*-dimensional spaces into three principal eigenvectors or axes. It is in the nature of PCA that the principal eigenvectors explain “most,” rather than “all,” of the variance. Hence there is a residual level of variance which may create a low level of statistical dependency between the primary axes. As the example of functional holography shows, however, this is not a barrier to projecting features into the three-dimensional space and using these projections to reveal hidden structures and relationships. The BBM follows the traditions set by phase spaces, functional holography, and other allied approaches. It reduces complexity by collapsing, or clustering, an *N*-dimensional space into those which explain the majority of variance in systems complexity; it turns typological scales into numeric, taxonomic scales using fuzzy logic; and it projects data into the three-dimensional space in order to reveal interactions between the key dimensions of interest (Walker et al., 2009). So whilst this offers a structured and somewhat comprehensive means to model complexity, the rating of problems across the three attributes does not account for any potential relationship between the attributes. For example, using the BBM problem rating process, it is possible to rate dynamism at 0.1 and uncertainty at 1 for a given problem. Whilst a robust problem analysis would unlikely produce such extreme output scores, in doing so it may prompt reflection on the part of analysts using the tool. Can a problem or system “really” have very low scores for multiplicity and very high scores for dynamism? Perhaps, but it is likely to be unusual and worthy of further scrutiny.

The authors argue for the orthogonal nature of the axes while at the same time acknowledging the possibility of some residual statistical interdependence. However, maintaining the assumption of statistical independence between the three attributes which comprise the spaces presented by the BBM has a practical benefit; it configures the problem space in a manner that can capture nuanced system properties. It allows for systems scoring at any points along the attribute scales to be modeled more granularly than if statistical dependence between attributes was assumed. For instance, it could be argued that a relatively simple task involving a large team of distributed agents in a tightly controlled environment, for example, maintenance work taking place within a routine rail track possession, may represent a work system which exhibits low dynamism, low uncertainty but high multiplicity. To illustrate this problem in context of the above example, a positive statistical correlation between multiplicity (which would likely be rated highly for this example) and the other attributes would produce a final set of fuzzy inputs reflecting a problem characterized by higher degrees of uncertainty and dynamism than may actually be present. In reality, the tightly controlled nature of a track maintenance possession and the detailed planning undertaken prior to its implementation represent a work system characterized by relatively low levels of uncertainty. In this example, the integration of positive statistical correlations between the attributes would produce a problem rating output that may be less reflective of the work system’s properties resulting in the BBM output recommending methods with lower PE against the observed problem attributes. In essence, whilst some degree of correlation between the three attributes may exist, integrating this relationship into the BBM utility space could impair key distinctions between system properties, leading to a “coarser” analysis output. The trade-off is the increased reliance on the analyst teams’ domain knowledge and their comprehension of the system tenets as they pertain to the given problem under analysis. Again, by making the tool open access this tension can be resolved through collective agreement more widely across the discipline, coupled with more robust sensitivity and reliability analyses.

## Conclusion

If one of the leading introductory texts in HF says of methods selection that “it depends” (Salvendy, 2012) then we have to ask two questions: first, does the phrase, which is largely correct, really suffice for a discipline seeking to have a bigger impact on societies’ grand challenges? Second, what does it actually “depend” on? According to the literature this question is one of the most problematic challenges encountered by HF practitioners today (Wickens et al., 2015). Despite this, answers in the literature are conspicuous by their relative absence. This would not be a problem were it not for the fact that

- So many HF methods exist and there is no clear guidance on which one of the hundreds should be applied and when (Stanton et al., 2013);
- The rate at which complex sociotechnical systems are becoming the focus of HF practice is outpacing the uptake of existing systemic methods and the development of new ones, and;
- The strategic direction of the discipline is driving us into areas of the existing HF utility space that are poorly served, if served at all, by the available suite of methods (Salmon, Walker et al., 2017).

The need to visit, let alone revisit, HF methods selection is long overdue. We argue that HF method selection is not well served by legacy and preference, by far the most common approach to method selection, and neither is it well served by the paucity of decision-support meta-methods, or even by method integration and triangulation. Valid attempts at the two former options have been made, but they remain poorly developed at present.

A more robust approach is presented in this paper, along with an attempt at a clear vector between theory and practice. The notion of PE sits at the theoretical end of the research/practice vector; the expedient need to acquire maximum insight for minimum analytical effort lies at the other. Connecting these points is a fuzzy logic approach for rating problems and methods on their inherent complexity attributes and the creation of defined problem, method, and utility spaces for calculating the match between “method” and “problem” in a very direct way. Two key insights are acquired, one conceptual and the other practical.

The conceptual insight is that out of 66 HF methods, rated using the complexity criterion and the fuzzy logic approach, nearly 80% fell within a comparatively narrow region of the problem space. Even by making a range of quite generous assumptions it is clear that certain regions of the HF problem space are either not well served with methods or not served at all. At present, if an HF problem is described by, assumed to be, or constrained to, low rates of dynamism, multiplicity, and uncertainty then there is no shortage of methods. If, however, one’s HF problem is characterized by high rates of dynamism, multiplicity, and uncertainty, and the client requires insights which confront these features overtly, then the availability of methods is much more limited, in some cases completely absent. An important implication is either that further, more sophisticated HF methods may require development or HF needs to connect with other disciplines whose methods provide such capacity (e.g., computational modeling approaches such as systems dynamics and agent-based modeling).

The second insight is that for practitioners not wedded by legacy or preference to a particular method, the approach described in this paper creates the chance to broaden methodological horizons considerably. The fuzzy logic ratings are of course open to reinterpretation and we actively welcome their wider use within the HF community at large. Moreover, we recommend that further study be undertaken to validate the BBM and that case studies and success stories be documented. An important goal of this approach is not just the output, but also the process of deriving it. The BBM approach is a way to think and reason about complexity, to build consensus about what it really means, and to discover new ways to tackle it. To that end the simple spreadsheet needed for practitioners to explore method selection and problem and method ratings sheets are included as Supplementary Material. If the strategic direction of the discipline becomes manifest in the years ahead then the value of HF will become increasingly recognized and the needs of clients will also become more sophisticated. As such, a satisfactory answer to the question “why did you choose that method?” will need to be provided. The BBM approach presented here enables “it depends” to be replaced with something more rigorous.

## Key Points

- HF practitioners have limited to guidance for HF method selection. This is becoming increasingly problematic as systems within which HF problems are nested are becoming more complex. Robust analysis of complex problems thus requires the deployment of equally sophisticated methods.
- There is currently no guidance to help HF practitioners select appropriate methodology for tackling HF problems nested in these systems.
- The BBM, a method selection framework, is presented with the primary aim of providing HF practitioners a robust framework to match HF methods to HF problems.
- The BBM provides fuzzy measures of system complexity based on the inherent system uncertainty, dynamism, and multiplicity, and provides users with a suite of methods best suited for efficient analysis in account of the measured system complexity.
- The secondary aim of the BBM is to encourage HF practitioners to broaden their methodological horizons for effective and appropriate HF analyses.
- Analysis of BBM method ratings indicates that HF practitioners are currently underserved with methods for tackling HF problems nested in complex systems. The implication of this finding is the requirement to develop sophisticated methodology that can be practically applied to HF problems nested in systems of increasing complexity.

## Supplemental Material

Supplementary Material 1 - Supplemental material for The Binary-Based Model (BBM) for Improved Human Factors Method Selection Click here for additional data file. Supplemental material, Supplementary Material 1, for The Binary-Based Model (BBM) for Improved Human Factors Method Selection by Matt Holman, Guy Walker, Terry Lansdown, Paul Salmon, Gemma Read and Neville Stanton in Human Factors: The Journal of Human Factors and Ergonomics Society

Supplementary Material 2 - Supplemental material for The Binary-Based Model (BBM) for Improved Human Factors Method Selection Click here for additional data file. Supplemental material, Supplementary Material 2, for The Binary-Based Model (BBM) for Improved Human Factors Method Selection by Matt Holman, Guy Walker, Terry Lansdown, Paul Salmon, Gemma Read and Neville Stanton in Human Factors: The Journal of Human Factors and Ergonomics Society
