# Supplementary material for: The Binary-Based Model (BBM) for Improved Human Factors Method Selection
Source: Hum Factors. 2020 Jun 18;63(8):1408–36. doi: 10.1177/0018720820926875 (PMC8593309; doi:10.1177/0018720820926875)
Supplement: Supplementary Material 2 - Supplemental material for The Binary-Based Model (BBM) for Improved Human Factors Method Selection [file sj-doc-2-hfs-10.1177_0018720820926875.doc]

**Problem Ratings**

| **Dynamism** | | |
| --- | --- | --- |
| **System Tenet** | **Score** | **Decision Log** |
| Emergence - To what extent can an outcome or property that is a result of the interactions between components in the system not be fully explained by examining the components alone? |  |  |
| Decrementalisation - To what extent can small changes in normal performance gradually result in large changes? |  |  |
| Performance Variability - To what extent do systems and components change performance and behaviour to meet the conditions in the world and environment in which the system must operate? |  |  |
| Contribution of the Protective Structure - To what extent does the protective structure of system control that are intended to optimise the system, instead do the opposite?  -Does the protective structure inhibit performance variability?  -Does it introduce or impose new tasks that do not contribute to the goal?  -Are unnecessary controls introduce or imposed? |  |  |
| **Fuzzy Input (Average)** |  |  |

| **Uncertainty** | | |
| --- | --- | --- |
| **System Tenet** | **Score** | **Decision Log** |
| Unruly Technology - To what extent does technology introduce and sustains uncertainties about how and when things may fail? |  |  |
| Sensitivity to Initial Conditions – To what extent can seemingly negligible changes in initial conditions affect overall system behaviour? |  |  |
| Non-linear Interactions - To what extent can/do interactions and relationships between components produce unpredictable outcomes? e.g. Inconsequential things have large effects; cannot predict the effect of changes |  |  |
| Inadequate Feedback Loops - To what extent is the communication structure and information flow to evaluate control requirements of hazardous processes inadequate? |  |  |
| **Fuzzy Input (Average)** |  |  |

| **Multiplicity** | | |
| --- | --- | --- |
| **System Tenet** | **Score** | **Decision Log** |
| Modularity - To what extent does normal performance depend on the interaction of an organisation of subsystems and components which are designed and operate largely independently of each other? |  |  |
| Vertical Integration - To what extent do multiple interaction between levels in the system hierarchy degrade communication/actions/decisions within the system? |  |  |
| Constraints - To what extent are there multiple system constraints which cause or influence limitations in the behaviours available to components within a system? |  |  |
| Functional dependencies - To what extent does the system exhibit multiple dependencies between system components that are not wanted or expected?  E.g. Losing communications due to poor signal |  |  |
| **Fuzzy Input (Average)** |  |  |

**Methods Ratings**

| **Dynamism** | | |
| --- | --- | --- |
| **System Tenet** | **Score** | **Decision Log** |
| Emergence - To what extent can the method capture outcomes or properties that are a result of the interactions between components in the system not be fully explained by examining the components alone? |  |  |
| Decrementalisation - To what extent the method account for small changes in normal performance which gradually result in large changes? |  |  |
| Performance Variability - To what extent can the method account for changing systems and components performance and behaviour as the system meets the conditions in the world and environment in which it must operate? |  |  |
| Contribution of the Protective Structure - To what extent can the method account for how the protective structure of system control that is intended to optimise the system, may instead do the opposite?  - Can it account for the protective structure inhibiting performance variability?  - Can it be used to understand how the protective structure may introduce or impose new tasks that do not contribute to the goal? |  |  |
| **Fuzzy Input (Average)** |  |  |

| **Uncertainty** | | |
| --- | --- | --- |
| **System Tenet** | **Score** | **Decision Log** |
| Unruly Technology - To what extent can the method account for the uncertainty introduced and sustained by technology? |  |  |
| Sensitivity to Initial Conditions – To what extent can the method capture and analyse how seemingly negligible changes in initial conditions may affect overall system behaviour? |  |  |
| Non-linear Interactions - To what extent can the method be used to model interactions and relationships between components that produce unpredictable outcomes? |  |  |
| Inadequate Feedback Loops - To what extent can the method capture and analyse the communication structure and information flow to evaluate control requirements of hazardous processes? |  |  |
| **Fuzzy Input (Average)** |  |  |

| **Multiplicity** | | |
| --- | --- | --- |
| **System Tenet** | **Score** | **Decision Log** |
| Modularity - To what extent can the method model performance as a result of the interaction of an organisation of subsystems and components which are designed and operate largely independently of each other? |  |  |
| Vertical Integration - To what extent can the method evaluate the impact of multiple interaction between levels in the system hierarchy and any subsequent degradation of communication/actions/decisions within the system? |  |  |
| Constraints - To what extent can the method evaluate the impact of multiple system constraints which cause or influence limitations in the behaviours available to components within a system? |  |  |
| Functional dependencies - To what extent can the method capture multiple dependencies between system components that are not wanted or expected?  E.g. Losing communications due to poor signal |  |  |
| **Fuzzy Input (Average)** |  |  |
